# Connections Between Endoplasmic Reticulum Stress and Prognosis of Hepatocarcinoma

**DOI:** 10.3390/bioengineering11111136

**Published:** 2024-11-11

**Authors:** Ming Wu, Jinxing Yan, Shimei Qin, Lei Fu, Shibin Sun, Wan Li, Junjie Lv, Lina Chen

**Affiliations:** College of Bioinformatics Science and Technology, Harbin Medical University, Harbin 150081, China; wm734393188@163.com (M.W.); yanjinxing0712@163.com (J.Y.); shimeiqin1128@163.com (S.Q.); fulei@hrbmu.edu.cn (L.F.); sunshibin22@163.com (S.S.); liwan@hrbmu.edu.cn (W.L.)

**Keywords:** endoplasmic reticulum stress, hepatocellular carcinoma, prognostic model, high-risk

## Abstract

Endoplasmic reticulum (ER) stress is a state in which misfolded or unfolded proteins accumulate in the lumen of the ER as a result of some exogenous or endogenous factors. It plays a crucial role in the pathogenesis of malignancies, affecting cell survival, proliferation, and metastasis in cancer. ER stress genes could provide new ideas for potential therapeutic targets in cancer. In our study, we aimed to construct an ER stress-related genes (ERGs) model for hepatocellular carcinoma (HCC). ERGs with differential expression and significant survival were screened to construct a prognostic model. The effectiveness of the model was successfully validated by external datasets. High and low-risk groups were classified based on risk scores. Functional analysis showed risk groups involved in the unfolded protein response, DNA repair, and other differential pathways. When compared to patients with low risk, the prognosis for HCC patients in the high-risk group might be worsened by disruptions in these pathways. Importantly, we considered genomic druggability and predicted drugs. Sorafenib-induced autophagy in HCC cells through an ES stress mechanism. Sorafenib was more sensitive for high-risk patients. In brief, our model predicted the prognosis of HCC and provided novel treatment strategies for the study of other cancers.

## 1. Introduction

The endoplasmic reticulum (ER) is one of the multifunctional organelles in eukaryotic cells, within which protein synthesis, processing and transporting lipid and steroid synthesis, and calcium storage all take place [1,2]. Endoplasmic reticulum stress (ERS) is affected by numerous genetic and environmental factors and is characterized by a buildup of unfolded or misfolded proteins [3].

Cells have evolved protein quality-control mechanisms that act on a variety of pathways, including the unfolded protein response (UPR), ER-associated degradation (ERAD), and autophagy to maintain ER homeostasis [4]. UPR is predominantly coordinated by three ER-resident transmembrane proteins as sensors, namely activating transcription factor 6 (ATF6), PRKR-like ER kinase (PERK), and inositol-requiring enzyme 1 (IRE1), which properly reduces the load of unfolded proteins to restore the cell homeostasis [5,6]. ERAD is in charge of removing misfolded proteins in the ER so that cytosolic proteasomal enzymes can degrade them [4,7].

Any cancer results from a variety of genetic, transcriptional, translational, and metabolic abnormalities that create an unfavorable microenvironment that leads tumor cells to experience constant ERS, which ultimately affects how well they function, what happens to them, and whether they survive [5,8]. ERS may lead to unregulated cell proliferation by enhancing the protein folding capacity and prolonging resistance to anticancer drugs [9]. Chronic ERS is directly involved in the formation of hepatocellular tumorigenesis [10]. It plays an important role in the proliferation, invasion, metastasis, angiogenesis, and drug resistance of HCC, providing a theoretical basis for anti-HCC strategy [11,12]. Certain pharmacological ERS-inducing agents have the potential to be anticancer therapies [11]. 

There is a dismal prognosis in HCC with cancer cells suffering from ERS [13]. Prognostic prediction of HCC can guide clinical practice [14]. Consequently, we started with the ERGs to examine the mechanism of ERS in cancer and explore the prognostic markers of liver cancer.

In an effort to identify potential therapeutic targets that could be utilized to treat or prevent HCC, we concentrated on the investigation of genetic and clinical aspects. In this research, on the basis of differentially expressed ERGs (DE-ERGs) in the TCGA-LIHC dataset, we first carried out a survival analysis. Additionally, with the help of the TCGA and ICGC databases, we successfully developed and validated a risk model for ERGs to forecast the outcomes of HCC patients. The effectiveness of the risk score was then examined. Importantly, we explored the pathways connected to risk differential genes. Furthermore, we analyzed genomic druggability and forecasted medicines. These findings might offer a fresh perspective on HCC survival forecasting and treatment approaches.

## 2. Materials and Methods

### 2.1. Data Selection

RNA-seq and clinical information of HCC patients were obtained from the TCGA data portal (TCGA-LIHC cohort-FPKM) (http://portal.gdc.cancer.gov/, accessed on 21 October 2022) as training sets. RNA-seq data included 424 cases, 374 of which were tumor samples and 50 adjacent normal samples. ICGC-LIRI-JP was selected as the verification cohort from the International Cancer Genome Consortium (ICGC) database (https://dcc.icgc.org/, accessed on 4 November 2022). 

### 2.2. Collection of ERGs

We collected ERGs from two databases. The KEGG PATHWAY and MsigDB (Molecular Signature Database, https://www.gsea-msigdb.org/gsea/msigdb/index.jsp, accessed on 16 September 2022) databases [15] were among the most widely used and comprehensive functional databases for gene set enrichment analysis, including ERS-related pathways. An amount of 171 ERGs within a pathway (protein processing in the endoplasmic reticulum—Homo sapiens (human) were extracted from the KEGG PATHWAY Database (https://www.genome.jp/kegg/pathway.html, accessed on 25 September 2022). Alternatively, the 144 ERGs involved in the IRE1, PERK, ATF6, and ERAD pathways were collected from MsigDB. Studies have shown that these four pathways play a pivotal role in the process of ERS response [5]. In total, 247 ERGs from the KEGG pathway database (171 ERGs) and the MsigDB database (144 ERGs) were used for subsequent analysis.

### 2.3. DE-ERGs Analysis

To choose ERGs that contribute to the development and progression of cancers, differentially expressed genes (DEGs) between tumor tissues and normal tissues were analyzed using the “limma” package. It included the following steps: (a) Loading the data, (b) fitting linear models for differential expression, (c) performing differential expression analysis using comparative analysis and the Empirical Bayes method, and (d) generating results like fold changes, *p*-values, and adjusted *p*-values. See the Code S1 for detailed R codes. The DEGs with adjusted *p*-value < 0.05 and |log2 (fold change)| > 1 were considered to meet the screened criterion. DE-ERGs are ERGs (mentioned above) included in differential genes.

### 2.4. Development of a Prognostic Model with DE-ERGs

The univariate Cox regression analysis was used to identify DE-ERGs related to survival with *p*-values < 0.05. Then, the significant genes were selected for the least absolute shrinkage and selection operator (LASSO) Cox regression, which was performed by using the “glmnet” R package(4.3.0). We established LASSO-Cox regression based on the training dataset to select prognostic variables for OS evaluation with the following procedure concretely: (1) the parameter *λ* was the shrinkage parameter and determined the extent of variable selection, with larger values corresponding to a larger penalty and a greater number of variables removed. Smaller values of the cross-validation (CV) error were expected to be better at expressing the generative model of the data. The minimum, if it existed, of the CV error when changing *λ* was thus considered to obtain an optimal value of *λ*. The optimal value of the penalty parameter corresponding to *λ* in LASSO was chosen by performing 10-fold cross-validation; (2) the selected lambda (*λ*) was determined by the smallest 10-fold cross-validation based on partial-likelihood deviance; (3) those selected variables with non-zero coefficients dependent on their information characteristics by LASSO were used for multivariable Cox regression analysis.

After 1000 repetitions of the algorithm, model genes with frequencies greater than 700 times were chosen to further calculate the risk score (RS). Pearson correlation was used to describe the co-expression states of genes. As a result, a model containing prognostic genes was created. The RS for each patient was calculated as the sum of the expression level of a model gene multiplied by its corresponding regression coefficient. The formula used to calculate the RS is presented in Equation (1). In Equation (1), *n* denoted the number of model genes, *Expi* represented the expression value of the ith genes, and weight *Coefi* represented the coefficient of each gene. The formula was as follows:

(1) $$\mathrm{RiskScore}=\sum_{i=1}^{n}Coefi\times Expi,$$

Given the median value of the RS, the clinical samples were classified into high-risk and low-risk groups. Thereupon, model genes were analyzed using multivariate Cox and Kaplan–Meier (KM) curves. A *p*-value < 0.05 was considered statistically significant.

### 2.5. Model Reliability Evaluation

The independent predictive efficiency of the prognostic signatures involving RS was evaluated by univariate and multivariate Cox analyses. The hazard ratio (HR) and 95% confidence intervals (CI) were calculated. The KM survival curve was applied to estimate the predictive ability of the risk group. The time-dependent receiver operating characteristics (ROC) curve was generated to analyze the 1-year, 3-year, and 5-year specificity and sensitivity of the RS [16]. A support vector machine (SVM) classifier was built to distinguish between high-risk and low-risk samples [17,18]. Leave-one-out cross-validation (LOOCV) was used to evaluate the overall performance of the classifier. Then, the value of the area under the curve (AUC) was calculated to evaluate the classification performance.

### 2.6. OncoPredict for Drug Sensitivity Analysis

The OncoPredict R package was employed to calculate drug sensitivity for cancer samples from the TCGA-LIHC dataset. The OncoPredict used the gene expression profile and half-maximal inhibitory concentration (IC50) of the cancer cell lines to drugs from CTRP and GDSC (training dataset) to build ridge regression models. The IC50 value of each drug in each sample from the TCGA-LIHC cancer dataset was assessed with the training set via the “oncoPredict” package, representing the potential drug response in that patient. The sensitivity of the drugs (between the high- and low-risk groups) was analyzed using Wilcoxon rank sum tests. The *p* < 0.05 was set as the threshold for significance.

## 3. Results

### 3.1. Prognostic Endoplasmic Reticulum Stress Model of HCC

**Figure 1:**
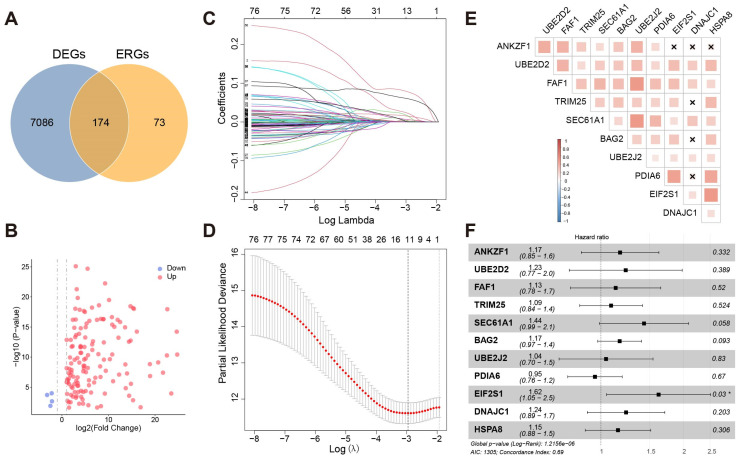
(**A**) The intersection of differential genes and ERGs. (**B**) Volcano map visualized differential ERGs in HCC. Red means upregulated genes, and blue means downregulated genes. (**C**) LASSO coefficient profiles of the 79 hepatocellular prognosis genes. (**D**) Ten-time cross-validation for tuning parameter selection in the LASSO model. The solid vertical lines are partial likelihood deviance ± standard error (SE). The dotted vertical lines are drawn at the optimal values by minimum criteria and 1-SE criteria. A *λ* value of 0.052 with log(*λ*) = −2.956 was chosen by 10-time cross-validation via min criteria. (**E**) Co-expression of eleven genes. “×”: *p* > 0.05. (**F**) Multivariate Cox regression analysis of eleven model genes. “*”: *p* <= 0.05.

First, 247 ERGs were collected strictly in total (Supplementary Table S1). Furthermore, 7260 DEGs were selected in 374 tumor samples and 50 normal samples from the TCGA-LIHC cohort. Next, we identified 174 DE-ERGs (Figure 1A). It followed that most ERGs were highly expressed in HCC (Figure 1B).

First, univariate Cox regression was used to investigate the relationship between the expression levels of 174 DE-ERGs and survival. Using the cut-off threshold of Cox *p* < 0.05, 79 DE-ERGs related to overall survival (OS) were screened as potential prognostic genes. Subsequently, these prognostic genes were checked in the 1000-times-repeated LASSO-Cox regression algorithm for feature selection and reduced overfitting of the model, and 11 genes were identified with the lambda of 0.052 with log(*λ*) = −2.956 (Figure 1C,D). Pearson’s analysis displayed that the expression of these genes had lower correlations (*p* < 0.05, −0.5 < *r* < 0.5) (Figure 1E). This suggested that they could act as independent factors. 

The 11 genes with non-zero coefficients in the LASSO regression model were chosen to further calculate the RS, including ANKZF1, UBE2D2, FAF1, TRIM25, SEC61A1, BAG2, UBE2J2, PDIA6, EIF2S1, and DNAJC1. The RS of the prognostic model was calculated using the following formula:

*RiskScore* = 0.0229 × *Exp*_ANKZF1_ + 0.0060 × *Exp*_UBE2D2_ + 0.0046 × *Exp*_FAF1_ + 0.0163 × *Exp*_TRIM25_ + 0.0016 × *Exp*_SEC61A1_ + 0.0783 × *Exp*_BAG2_ + 0.0030 × *Exp*_UBE2J2_ + 0.0018 × *Exp*_PDIA6_
+ 0.0603 × *Exp*_EIF2S1_ + 0.0151 × *Exp*_DNAJC1_ + 0.0003 × *Exp*_HSPA8_, (2)

The correlation between model genes and the prognosis of HCC was further examined by multiple factors regression analysis (Figure 1F). Among the eleven genes, ten genes were classified into risky groups with HR > 1 related to poorer prognosis, whereas one remaining gene (PDIA6) was regarded as a low-risk gene with HR < 1. In addition, nine genes were statistically significant results in the KM survival analysis except for TRIM25 and DNAJC1 (Supplementary Figure S1).

### 3.2. The Risk Score Serves as an Independent Prognostic Indicator

**Figure 2:**
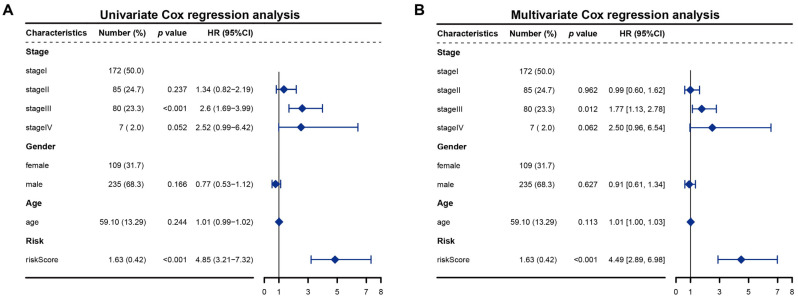
(**A**) Univariate Cox regression analysis of RS and clinical variables; (**B**) Multivariate Cox regression analysis of RS and clinical variables.

Univariate and multivariate regression analyses were performed to determine if the risk score prognostic model is an independent prognostic factor for HCC. The RS and clinical-pathological parameters (including clinical stage, sex, and age) were analyzed. In the univariate as well as multivariate Cox regression models, RS met *p* < 0.05 and HR > 1 (Figure 2A,B). The RS was proved to be an independent prognostic indicator and risk factor. These findings demonstrated that the risk score prognostic model was reliable in forecasting the survival of patients with HCC.

### 3.3. Identification and Validation of Clinical Risk Indicators

**Figure 3:**
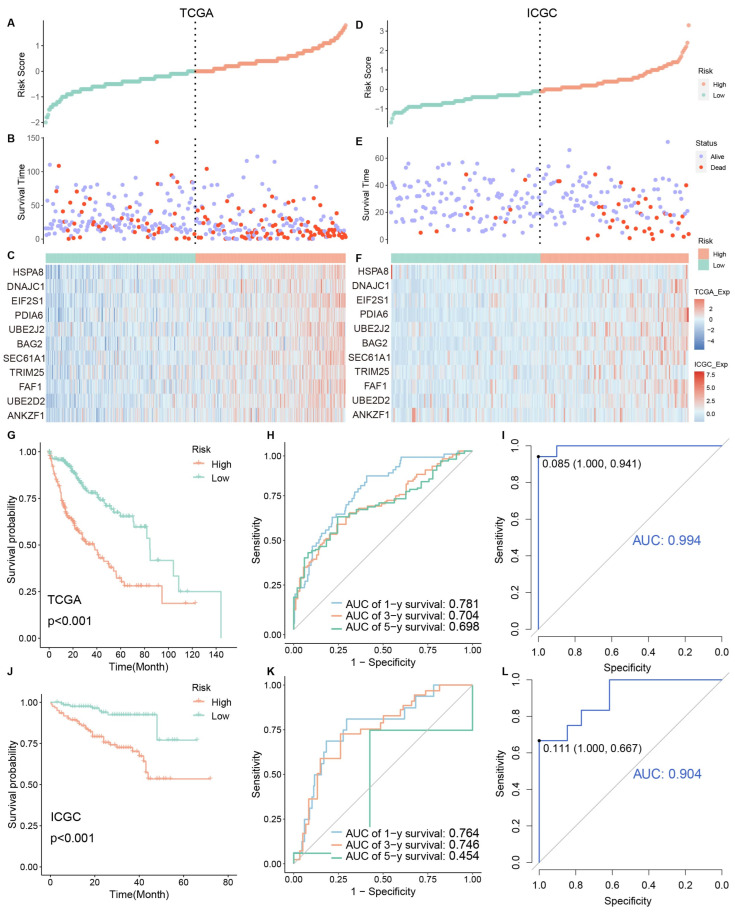
(**A**–**F**) Risk factor association maps show the RS, survival status, and expression distribution of genes. The left side is the training set, and the right side is the verification set. (**G**,**J**) KM curves of high and low-risk groups. (**H**,**K**) ROC curves to predict the sensitivity and specificity of 1-, 3-, and 5-year survival, according to the RS. (**I**,**L**) ROC curve of model genes in high and low-risk groups. (**G**–**I**) Training set data. (**J**–**L**) verification set data.

We ranked patients’ risk scores in the TCGA cohort and analyzed their distribution (Figure 3A–C). The samples were assigned to the high-risk group (*n* = 184) or low-risk group (*n* = 184) according to the median value of the RS. The survival status of HCC patients was marked on the dot plot. With increasing the RS, the number of dead patients increased. The heatmap revealed the expression distribution of eleven ERGs between two different risk groups. Their expression was higher in the high-risk group. 

In addition, the KM curve indicated that the patients in the high-risk group exhibited worse OS than those in the low-risk group (*p* < 0.001) (Figure 3G). To assess the accuracy of the prognostic model, we also performed a time-dependent ROC curve. The AUC was 0.781, 0.704, and 0.698 for the 1, 3, and 5-year survival times, respectively, indicating that this eleven-gene prognostic model performed well as a predictor of OS (Figure 3H). Expression values of model genes were used as features for SVM classifiers to distinguish between risk samples (Figure 3I). They had high classification performance (AUC > 0.95).

To validate the constructed prognostic model, the predictive performance of the model on the training set was further evaluated. ICGC-LIRI-JP, an external dataset containing 243 tumor samples, was used to study the predictive power of the developed risk model (Figure 3D,E). Analyzing the RS plot results confirmed that samples characterized by poor OS exhibited high risk. The expression of 11 selected genes was shown in the heatmap. Consistent with previous results, the low-risk group’s OS was better than that of the high-risk group (Figure 3J). The AUCs for 1, 3, and 5-year risk scores were 0.764, 0.746, and 0.454, respectively (Figure 3K). The 5-year AUC result was poor. In addition, all model genes could distinguish between high and low-risk samples with good performance (AUC > 0.75) in the verification set (Figure 3L). These results collectively indicated that the prognostic model performed well as a predictor of OS in HCC.

### 3.4. Function Evaluation

**Figure 4:**
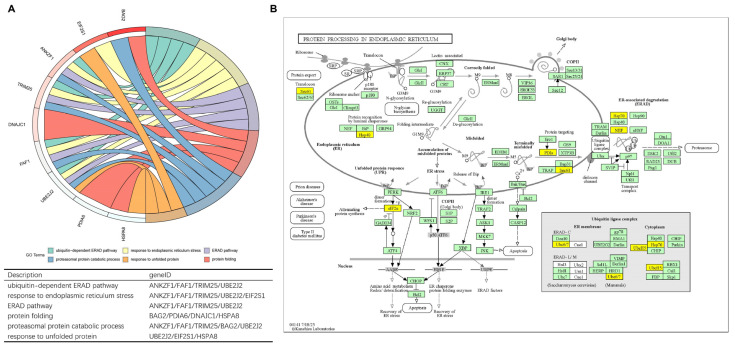
(**A**) The chord diagram visualizes the correlation between nine model genes and potential biological functions. (**B**) KEGG pathway maps for protein processing in endoplasmic reticulum-Homo sapiens (human) (hsa04141) where model genes are marked in yellow.

Model genes were subjected to Gene Ontology (GO) and Kyoto Encyclopedia of Genes and Genomes (KEGG) enrichment analysis by R package “clusterProfiler”. The corresponding chord diagram was plotted via the R package “GOplot”. Nine model genes were found in the Biological Process of GO enrichment analysis except for UBE2D2 and SEC61A1 (Figure 4A). UBE2J2, TRIM25, FAF1, and ANKZF1 participated in four pathways together, all related to the ERS and ERAD pathway; EIF2S1 was weightily involved in response to the ERS pathway. Eight model genes were discovered in the KEGG pathway (hsa04141), including all but ANKZF1, FAF1, and TRIM25 (Figure 4B). Most of the genes belonged to ubiquitin ligase complexes. Ultimately, EIF2S1 took part in the PERK-ATF4 signaling pathway indirectly resulting in attenuating protein synthesis and inducing autophagy [19]; HSPA8, BAG2, UBE2J2, and UBE2D2 participated in ERAD.

**Figure 5:**
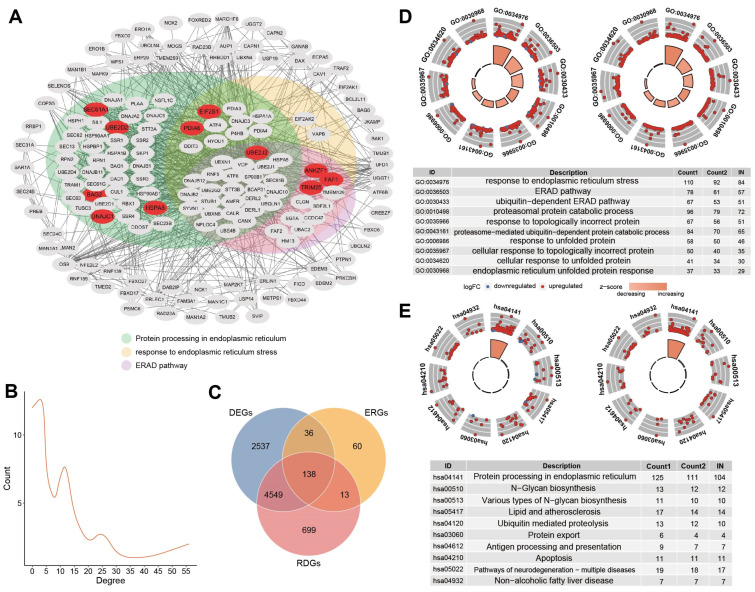
(**A**) PPI network of the 174 genes. The default required interaction score was set to a high confidence of 0.700. Model genes were marked in red. The background color of the dots represented the participation pathway. Green represented the hsa04141 pathway, yellow represented the pathway in response to ERS, and purple represented the ERAD pathway. (**B**) Degree distribution of nodes in the network. The number of nodes tended to decrease as the degree increased. (**C**) The intersection of ERGs, differential genes, and risk differential genes. The combined number of the three was 138, called the 138 gene set. (**D**) The figure on the left shows the GO enrichment of differential ERGs, and the figure on the right shows risk differential ERGs. (**E**) The figure on the left shows the KEGG enrichment of differential ERGs, and the figure on the right shows risk differential ERGs.

To analyze the role of model genes in tumorigenesis and the progression of HCC at a functional level, we used the STRING database to construct the PPI network of differential ERGs containing 11 genes. The STRING database (http://string-db.org, accessed on 27 July 2023) aims to provide a critical assessment and integration of protein-protein interactions, including physical as well as functional associations. The PPI networks of these 174 genes were mapped to better understand the correlation between the proteins encoded by them. There were 963 interaction relationships between 174 nodes, and most of them were involved in the KEGG ERS pathway (Figure 5A). UBE2J2-UBE2D2-TRIM25, DNAJC1-HSPA8-BAG2, and PDIA6-SEC61A1 had a reciprocal relationship in the model genes. SEC61A1, HSPA8, PDIA6, and UBE2D2 had a high degree of connectivity and played an important role in the network. Furthermore, risk differential ERGs (RDE-ERGs) were obtained based on high and low-risk groups, and model genes were RDE-ERGs as well. 138 ERGs were both differential genes and risk differential genes (Figure 5C). 

The results of GO analysis for these DE-ERGs and RDE-ERGs were presented, in which the most enriched terms were “response to endoplasmic reticulum stress”, “ERAD pathway”, and “response to unfolded protein”, respectively (Figure 5D). KEGG enrichment signaling pathways by these genes contained the most important “protein processing in endoplasmic reticulum” process, the “N-Glycan biosynthesis”, and the “ubiquitin-mediated proteolysis” (Figure 5E). 

Risk differential genes were involved in almost all aspects of ERS pathways (Supplementary Figure S2). First, risk differential genes (contain model gene: EIF2S1) spread across the PERK pathway. Secondly, MBTPS2 was the only non-risk differential gene in the ATF6 pathway. Thirdly, the IRE1 pathway contained two risk differential genes (TRAF2 and MAPK9). In short, all three pathways led to apoptosis. Finally, many risk differential genes were found throughout the ERAD pathway.

### 3.5. Functional Characterization Analysis of Cancer in High- and Low-Risk Groups

To investigate the potential function and significant pathways associated with the signature, the expression matrices of the different risk groups were imported and examined by the “GSVA” package. The differentially enriched pathways between the two groups mainly involved the unfolded protein response, protein secretion, PI3K signaling via AKT to mTORC1, DNA repair, E2F targets, MYC targets variant, and cell cycle G2/M checkpoint (Supplementary Figure S3). The GSVA results suggest that disturbances in these pathways may worsen the prognosis of HCC patients in the high-risk group compared to the low-risk group [20,21,22,23]. 

### 3.6. Druggability Analysis of ERGs

Given the importance of ERGs for HCC development and survival, the druggability of 138 genes was examined, which differed in both normal and disease as well as high and low-risk groups. Based on the public databases GDSC (Genomics Drug Sensitivity in Cancer) [24] and CTRP (Cancer Therapeutics Response Portal) [25,26], the responsiveness of targeted therapy in patients was assessed. A measure of the pharmacological reaction was illustrated: the half-maximal inhibitory concentration (IC50). It refers to the concentration of a drug required to inhibit half of a biological process (biological reaction with enzymes, receptors, cells, etc.). By measuring the response values of multiple concentration points, the inhibition curve was fitted by nonlinear regression analysis, and the IC50 value was calculated [27,28]. IC50 was used to measure the ability of a drug to induce apoptosis; that is, the lower the value, the stronger the induction ability. To characterize the clinically applicable therapeutic implications of ERGs, we first obtained experimentally validated ERS protein-drug interactions annotated by DrugBank [29], involving 197 drugs and 138 genes encoding for ERS proteins. We predicted the IC50 value of each drug in each HCC sample from the TCGA dataset using the “oncoPredict” package [30]. 

**Figure 6:**
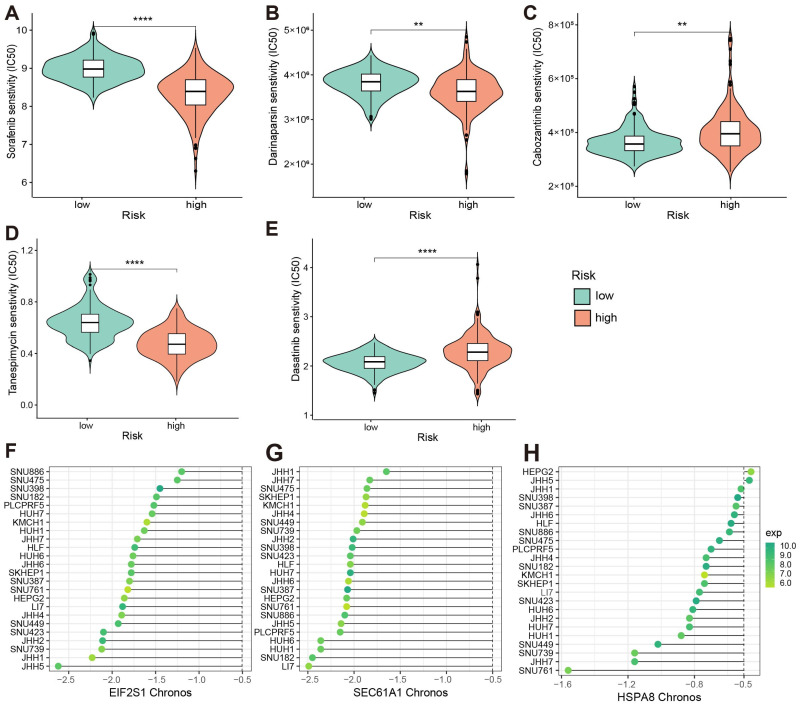
(**A**–**E**) Comparison of drug IC50 values in high- and low-risk populations. The lower the IC50 values, the more sensitive the drug. For the symbolic marking of statistical significance, “**”: *p* <= 0.01; “****”: *p* <= 0.0001. (**A**–**C**) The IC50 value of drugs targeting liver cancer; (**D**,**E**) The IC50 value of drugs targeting the 138 genes set; (**F**–**H**) Chronos values and expression values of model genes in different HCC cell lines. (**F**) EIF2S1; (**G**) SEC61A1; (**H**) HSPA8.

Comparing the drug sensitivity of patients with high- and low-risk populations, we found that different risk group patients exhibited differences with two drugs, including those of Tanespimycin (targeting HSP90AA1 and HSP90AB1 encoding proteins) and Dasatinib (targeting model gene HSPA8 encoding protein) (Figure 6D,E). It indicated that these two drugs may help in the treatment of HCC patients with high-risk scores. We furthermore obtained 89 drugs that have been traditionally used for the treatment of liver diseases. We observed significant differences in estimated IC50 values between the high-risk and low-risk groups for eight standard treatment drugs for liver diseases, including Sorafenib, Darinaparsin, Cabozantinib, Tacrolimus, Alvocidib, Ibrutinib, Regorafenib, and Lenvatinib. It was evident that in comparison to high-risk patients, low-risk patients displayed greater sensitivity to Cabozantini, while Sorafenib and Darinaparsin were the opposite (Figure 6A–E). It was suggested that standard treatment drugs for liver diseases might induce ERS in a way that they were not direct ER-targeting agents, but rather, it was through indirect or secondary means related to the drug’s primary mode of action. The results showed that perturbing ERGs could increase the susceptibility of cancer treatment drugs, becoming a potential target for HCC combination therapy.

For HCC patients, sorafenib was traditionally an effective first-line therapy [31]. Shi et al. [32] demonstrated that Sorafenib upregulated IRE1α signals in HCC cells, which in turn triggered autophagy. The Phase II study of Darinaparsin in patients with advanced HCC revealed that among the 15 patients, Darinaparsin was administered intravenously at a dose of 420 mg/m^2^, twice weekly with an interval of at least 72 h for 3 weeks within a 4-week cycle. When evaluated for toxicities, it was shown that the most common grade 1–2 toxicities could be safely managed, indicating that Darinaparsin was administered with tolerable toxicity profiles in HCC [33].

Essential genes in HCC were systematically determined using genome-wide screening of CRISPR-Cas9 derived from the DepMap portal. Candidate genes were defined as momentous genes with a Chronos score of <−0.5 across HCC cell lines (n = 24) [34]. As a result, a total of 53 ERGs were identified as essential genes whose gene knockout could inhibit the growth rate of HCC cell lines (Supplementary Table S2). The 85% of these genes were involved in ERS pathways (hsa04141). For example, the knockout of the HSPA8 gene could inhibit the growth rate of 22 out of the 24 (91.7%) HCC cell lines. Chronos values of model genes that met the criteria in different HCC cell lines are shown in Figure 6F–H. Furthermore, EIF2S1(model gene), NFE2L2, and ATF4 were located in the PERK pathway, MBTPS1 was located in the ATF6 pathway, TRAF2 was located in the IRE1 pathway, and there were seventeen genes (containing one model gene HSPA8) involved in the ERAD pathway. Fifteen genes (COPS5, DAD1, DDOST, EIF2S1(model gene), HYOU1, NPLOC4, PREB, PSMC6, RBX1, RPN1, SEC13, SEC61A1(model gene), SEC61G, SKP1, and VCP) met the criteria in all liver lineage. Furthermore, NPLOC4, RBX1, SKP1, and VCP participated in ERAD. Therefore, our result showed that inhibition of ERGs might be used as an antitumor therapy.

## 4. Discussion

Numerous parts of the world view HCC as a top contributor to cancer mortality. Even with the wide implementation of prevention, diagnosis, and treatment measures, the varied nature of tumor heterogeneity remains a major obstacle when it comes to accurately gauging patients’ prognosis. As a result, it is necessary to seek many clinical biomarkers of liver cancer. The evolution, aggressiveness, and treatment of HCC are known to be influenced by ERS [35,36]. Focusing on associated signaling pathways and related components of ERS might be crucial for fighting against HCC in recent years [12]. The study about HCC demonstrated that the expression level of many ERS markers (such as IRE1α, ATF6, PERK, and GRP78) was elevated, and they had an inverse relationship with the OS and clinicopathologic scores [37]. 

Bearing in mind the importance of ERS in cancer, an ERS model was formulated to predict the clinical value of HCC patients, the validity of the model was evaluated, and potential therapeutic agents were predicted. In order to investigate their relationship with survival and clinicopathological features, we therefore conducted a thorough and in-depth assessment of ERGs in addition to a retrospective analysis of HCC patients. A total of 79 DE-ERGs associated with OS were obtained as potential prognostic genes. A prognostic model was constructed using the 11 genes (ANKZF1, UBE2D2, FAF1, TRIM25, SEC61A1, BAG2, UBE2J2, PDIA6, EIF2S1, and DNAJC1) that were further screened. The expression level of HSPA8 and SEC61A1 significantly increased in HCC tissues compared to control tissues, and they had a poor prognosis. Survival analysis showed that ERGs could be used to predict the prognosis of patients. The RS was demonstrated to be an independent prognostic factor by Cox analysis. The model was able to effectively differentiate between high and low-risk groups of patients in terms of survival status. The validity of our model and model genes was confirmed using the ICGC database. Moreover, the SVM classifier, including model genes, was capable of effectively distinguishing HCC samples from healthy controls (AUC: 0.982) and yielded an AUC of 0.923 in an independent dataset (Supplementary Figure S4A,B).

Current reports on model genes about HCC were primarily derived from bioinformatics mining of public databases. Model genes had a 9/11 literature validation rate in relation to HCC on PubMed (https://pubmed.ncbi.nlm.nih.gov/, accessed on 7 September 2023). The prognostic validation rate of model genes associated with HCC was 10/11 on the HPA database (https://www.proteinatlas.org/, accessed on 29 May 2023).

The degree of aggressiveness and prognosis of liver cancer were closely correlated with elevated EIF2S1 expression levels. Patients with higher expression levels of EIF2S1 had worse prognoses. Overall, EIF2S1 could affect liver cancer’s invasion and metastasis ability [38]. Upregulation of HSPA8 and SEC61A1 affected hepatocarcinogenesis, identifying HSPA8 and SEC61A1 as potential therapeutic targets in liver cancer [39,40,41]. The results were consistent with our model. Yang et al. [42] viewed HSPA8 as a potential biomarker that could be utilized to accurately predict how immunotherapy would affect HCC. 

Additionally, the relativity between the eleven model genes and HCC was investigated by other databases (Comparative Toxicogenomics Database (CTD) and Human Gene Database (GeneCards)). The inference score reflected how closely CTD gene-disease networks and a scale-free random network resembled each other. The number of references related to liver cancer was counted. Disease relevance scores were calculated using the formula called Lucene’s practical scoring function in GeneCards. The higher the scores, the stronger the relationship. Genes with a relevance score ≥ 7 were deemed to be fine by GeneCards. EIF2S1, HSPA8, and SEC61A1 had high scores in both databases (Supplementary Figure S4C,D showed partial verification results).

The mechanism of model prognosis prediction was revealed by the functional enrichment of risk differential genes. In addition to ER-associated functional pathways, differential genes are enriched for pathways that affect cell survival and function, such as apoptosis, intrinsic apoptotic signaling pathway, and regulation of protein catabolic process.

Furthermore, to investigate the variations in signaling pathways among the risk groups, the GSVA results showed that the high-risk group was linked to tumor progression. The high-risk group exhibited activation of a large number of pathways closely related to tumor progression, as indicated by the results of KEGG Canonical Pathways gene sets, like biosynthesis, repair, and cell cycle. In contrast, low-risk patients were more likely to suffer several kinds of amino acid metabolism, such as tryptophan metabolism, tyrosine metabolism, glycine, serine and threonine metabolism, and several types of fatty acid metabolism, including butanoate metabolism, linoleic acid metabolism, arachidonic acid metabolism (Supplementary Figure S5A). Therefore, HCC progression might be influenced by the reprogramming of these metabolisms. Additionally, the GO enrichment analysis using C5 gene sets suggested that telomere maintenance was enriched in the high-risk group (Supplementary Figure S5B). Rebouissou and Nault [43] stated that the main genes whose mutations were implicated in HCC could be classified into six major biological pathways, including telomere maintenance, P53/cell cycle regulation, AKT/mTOR, oxidative stress, etc. It was consistent with the high expression pathway in the high-risk group. Several studies have shown that ERS is an important anti-cancer mechanism, including in liver cancer [12]. Preventing or treating HCC could be improved by relieving ERS as a novel, targeted, and successful treatment or prevention approach. Recently, there has been heightened interest in drugs that induce ERS as potential new anticancer drugs [44,45]. Given the importance of ERS for survival, drugs targeting ERG and HCC were screened at the DrugBank database, and IC50 values were used to determine the drug’s potential for cancer diagnosis or treatment. 

It is possible that ERS has both positive and negative aspects for cancer patients. The most straightforward and precise way to study the role of ERS in cancers is through the development of prognostic and diagnostic ERS models. Our findings are beneficial in developing some accurate and sensitive biomarkers for HCC, as the role of ERS in HCC is yet to be clarified. Even though we have validated this study using multiple angles and additional databases, certain limitations exist that need to be taken into account. We used a variety of analysis methods to comprehensively screen ERS prognostic model-related genes, including differential expression analysis, univariate Cox regression, and LASSO-Cox regression algorithm. However, with the publication of higher performance, higher precision big data methods, it may be important to consider other algorithms for screening genes to identify potentially prognostic molecules, such as machine learning algorithms. To guarantee its reliability, it is necessary to validate our model in sizable clinical samples and more external data. Second, it is still uncertain how specifically ERS regulates HCC. Third, with the continuous improvement of gene annotation functions, we will obtain more ER stress-related gene sets. Making full use of these resources will be of great significance for optimizing our model, gaining a deeper understanding of stress-related biological processes, and conducting more precise related research. In the future, our work investigates that how the proposed method can be extended to be applicable to the other types of datasets such as transcriptomics, proteomics, and metabolomics of cancer. In addition, preclinical pharmacological trials are unknown, which requires additional investigation. Our future plans involve continuing to explore deeply the mechanisms of ERGs and prognostic models in HCC and other malignancies on the basis of previous research.

## 5. Conclusions

In conclusion, we constructed a novel prognostic model containing eleven ERGs for HCC patients and verified its independent prognostic value. The model would help predict the prognosis of HCC and provide a new orientation to further research studies on the clinical prognosis of targeted ERS in HCC.

## Data Availability

The data that support the findings of this study are available upon request.

## Supplementary Materials

The following supporting information can be downloaded at: https://www.mdpi.com/article/10.3390/bioengineering11111136/s1, Table S1: Collected ERGs; Figure S1: KM survival curves for nine significant model genes; Figure S2: KEGG pathway maps; Figure S3: Pathway profile of HALLMARK; Table S2: 53 essential genes with Chronos scores less than –0.5 in HCC cell lines; Figure S4: (A,B) ROC curve of model genes for cancer and normal samples. (C,D) Correlation between model genes and liver cancer in external databases; Figure S5: Clustering heatmap illustrating the result of GSVA; Code S1: limma code.
