# Supplementary material for: Connections Between Endoplasmic Reticulum Stress and Prognosis of Hepatocarcinoma
Source: Bioengineering (Basel). 2024 Nov 11;11(11):1136. doi: 10.3390/bioengineering11111136 (PMC11591847; doi:10.3390/bioengineering11111136)
Supplement: Supplementary file 1 [file bioengineering-11-01136-s001.zip › bioengineering-3254805-supplementary.pdf]

Connections between endoplasmic reticulum stress and prognosis of Hepatocarcinoma

Ming Wu [1], Jinxing Yan [1], Shimei Qin [1], Lei Fu [1], Shibin Sun [1], Wan Li[1], Junjie Lv [1], Lina Chen [1,*] and Li Wang [1,*]

Supplementary Materials

Table S1

Collected endoplasmic reticulum stress genes.

**Endoplasmic reticulum stress genes**				
AMFR	EDEM2	NPLOC4	TMEM129	USP14
ANKZF1	EDEM3	OS9	TMEM259	USP19
AQP11	ERLEC1	PRKN	TMEM67	USP25
ATF6	ERLIN1	PSMC6	TMUB1	VCP
ATXN3	ERLIN2	RCN3	TMUB2	WFS1
AUP1	FAF1	RHBDD1	TOR1A	XBP1
BAG6	FAF2	RHBDD2	TRIM13	YOD1
BCAP31	FAM8A1	RNF103	TRIM25	ATF6B
BRSK2	FBXO17	RNF121	UBAC2	CREBZF
CALR	FBXO2	RNF139	UBE2G2	DDIT3
CALR3	FBXO27	RNF175	UBE2J1	MBTPS1
CANX	FBXO44	RNF185	UBE2J2	MBTPS2
CAV1	FBXO6	RNF5	UBE4A	AGR2
CCDC47	FOXRED2	RNFT1	UBE4B	BAK1
CLGN	GET4	RNFT2	UBQLN1	BAX
DERL1	HERPUD1	SDF2L1	UBQLN2	BCL2L11
DERL2	HM13	SEC61B	UBXN1	BFAR
DERL3	HSP90B1	SEL1L	UBXN10	COPS5
DNAJB12	HSPA5	SEL1L2	UBXN4	DAB2IP
DNAJB14	JKAMP	SELENOS	UBXN6	DDRGK1
DNAJB2	MAN1A1	SGTA	UBXN8	ERN1
DNAJB9	MAN1B1	SGTB	UFD1	ERN2
DNAJC10	MARCHF6	STT3B	UGGT1	FICD
DNAJC18	NCCRP1	STUB1	UGGT2	PARP16
ECPAS	NFE2L2	SVIP	UMOD	PTPN1
EDEM1	NGLY1	SYVN1	USP13	TMEM33

Endoplasmic reticulum stress genes				
UFL1	SIL1	SEC24D	DNAJC5	MMP24-AS1-EDEM2
VAPB	HYOU1	PDIA4	DNAJC5B	P4HB
ABCA7	DNAJB11	PDIA6	DNAJC5G	CKAP4
ATF4	DNAJC1	ERP29	HSP90AA1	RRBP1
BOK	DNAJC3	TXNDC5	HSP90AB1	STT3A
EIF2AK3	GANAB	ERO1A	HSPH1	MOGS
EIF2S1	PRKCSH	ERO1B	HSPA4L	MAPK10
NCK1	PDIA3	SSR1	BAG1	MAPK9
NCK2	MAN1A2	SSR2	BAG2	CAPN1
PPP1R15A	MAN1C1	SSR3	HSPBP1	CAPN2
PPP1R15B	LMAN2	SSR4	CRYAA	CASP12
PTPN2	LMAN1	TRAM1	CRYAB	UBE2G1
QRICH1	LMAN1L	TRAM1L1	PLAA	UBE2D4
TMED2	PREB	UBXN2A	RAD23B	UBE2D1
SEC61A1	SAR1A	NSFL1C	RAD23A	UBE2D2
SEC61A2	SAR1B	HSPA8	UBQLN3	UBE2D3
SEC61G	SEC13	HSPA1A	UBQLN4	RBX1
SEC62	SEC31A	HSPA2	ATXN3L	CUL1
SEC63	SEC31B	HSPA1L	EIF2AK1	SKP1
RPN1	SEC23B	HSPA1B	EIF2AK2	BCL2
RPN2	SEC23A	HSPA6	EIF2AK4	TRAF2
DAD1	SEC24B	DNAJA1	MAP3K5	
TUSC3	SEC24A	DNAJA2	MAP2K7	
DDOST	SEC24C	DNAJB1	MAPK8	

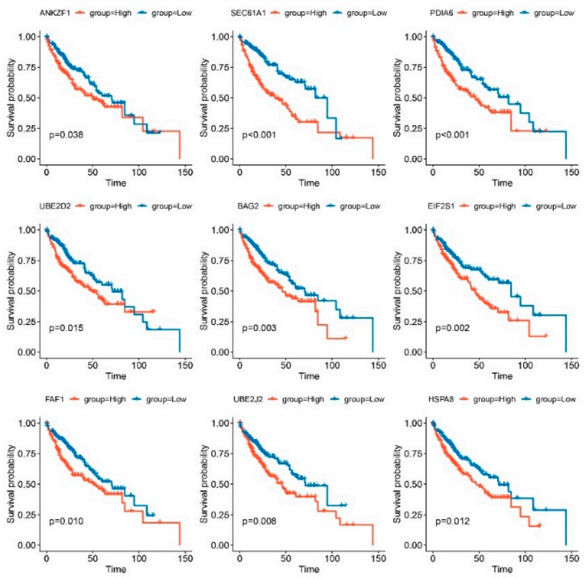

Figure S1

KM survival curves for nine significant model genes.

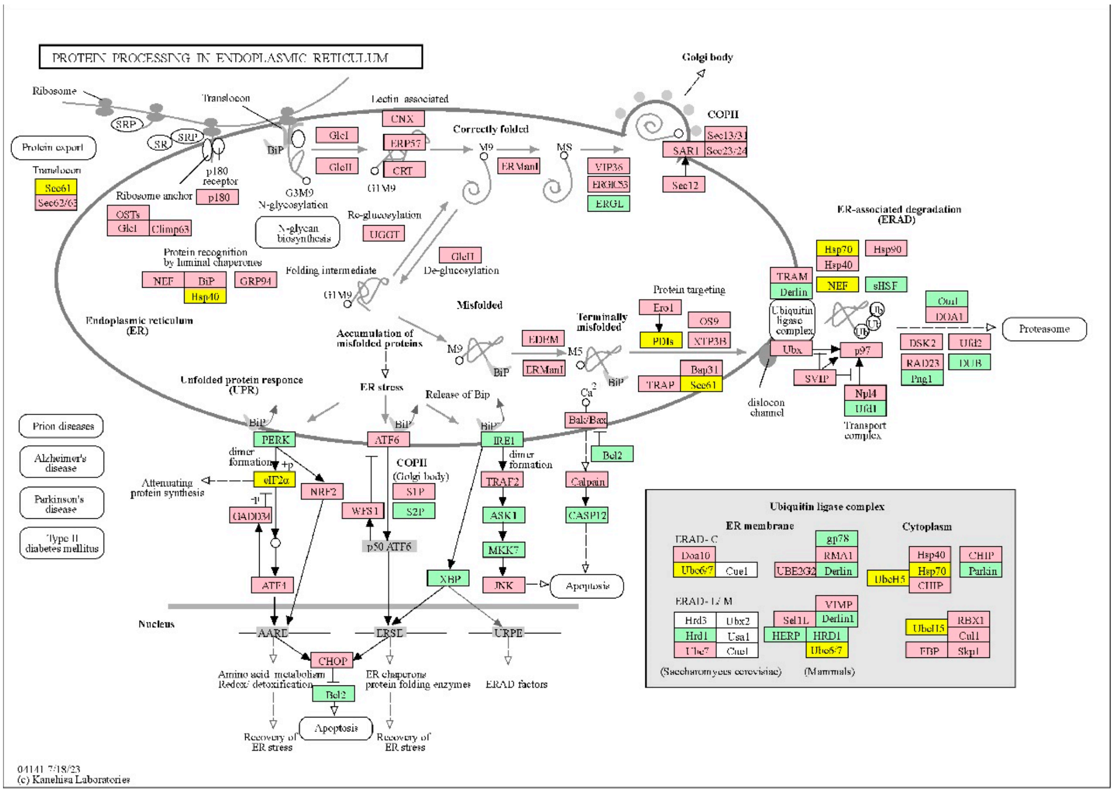

Figure S2

KEGG pathway maps for protein processing in endoplasmic reticulum-Homo sapiens (human)(hsa04141) where risk differential genes are marked in pink.

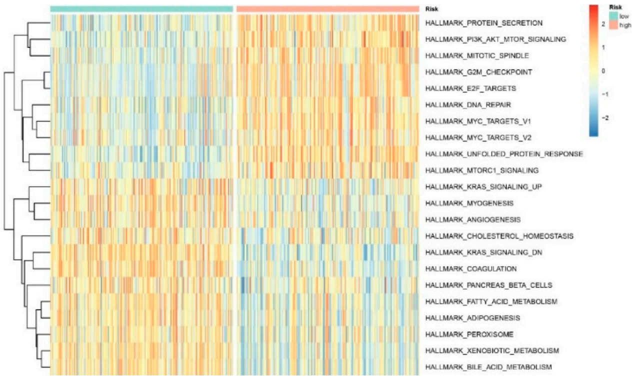

Figure S3

Pathway profile of HALLMARK. Rows represented biological pathways, and columns represented patients with HCC. Each grid represented a score of pathway activity calculated by GSVA. The upper horizontal bar marked patient information, including risk groups. GSVA-derived clustering heatmap of differentially expressed gene sets. Red, blue, and white respectively represent high

expression levels, low expression levels, and no expression differences among the genes.

Table S2

53 essential genes with Chronos scores less than -0.5 in HCC cell lines. Model genes are marked in red.

Essential genes					
ATF4	DAD1	HSP90AA1	NFE2L2	SEC13	STUB1
BAG6	DDOST	HSP90AB1	NPLOC4	SEC61A1	TMED2
BAK1	DNAJA1	HSP90B1	PREB	SEC61G	TRAF2
BCAP31	DNAJA2	HSPA5	PSMC6	SEC63	UBE2G2
CALR	DNAJB11	HSPA8	PTPN1	SKP1	UBE2J2
CAPN1	DNAJB12	HYOU1	QRICH1	SSR2	UBE4B
CCDC47	EIF2S1	MARCHF6	RBX1	SSR3	UBQLN4
COPS5	FAF2	MBTPS1	RPN1	STT3A	VCP
CUL1	GANAB	MOGS	RPN2	STT3B	STUB1

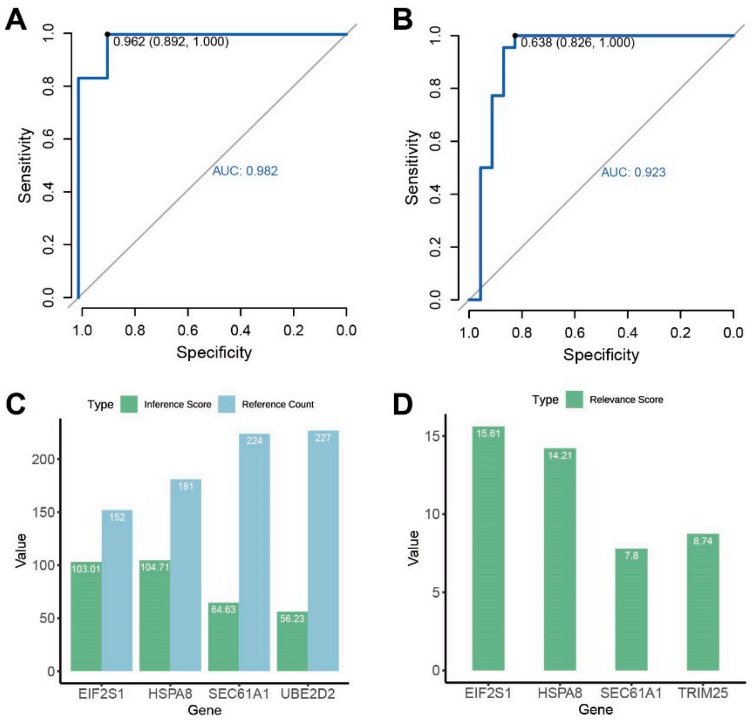

Figure S4

(A-B) ROC curve of model genes for cancer and normal samples. (A) Training set; (B) Validation set; (C) Correlation

between model genes and hepatocellular carcinoma in CTD Database; (D) Correlation between model genes and liver hepatocellular carcinoma in GeneCards Database.

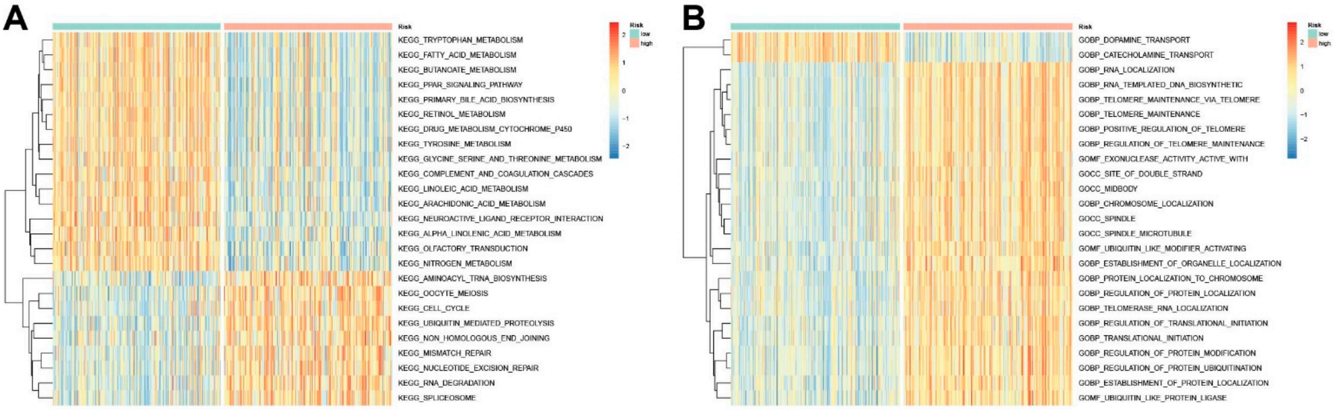

Figure S5

Clustering heatmap illustrating the result of GSVA. (A) Pathway profile of KEGG; (B) Pathway profile of GO.

Code S1

```
library(limma)#导入R包
exp<-read.csv("LIHC_PRE.CSV",row.names = 1) #表达谱数据
barcode<-substring(colnames(exp),14,15)#查看样本分组
table(barcode)
group_list <- ifelse(substring(colnames(exp),14,15)=="11","Normal","Tumor")#分组信息
design <- model.matrix(~0+factor(group_list))#生成模型设计矩阵
colnames(design)=levels(factor(group_list))
contrast.matrix<-makeContrasts("Tumor-Normal",levels = design)#构建比较矩阵
fit <- lmFit(exp,design) #线性模型拟合
fit2 <- contrasts.fit(fit, contrast.matrix) ##生成模型设计矩阵
fit3 <- eBayes(fit2) #差异分析
Output <- topTable(fit3, coef=1, n=Inf)#提取所有基因表格
Output = na.omit(Output)
Output$change = ifelse(Output$adj.P.Val< 0.05 & abs(Output$logFC) >=1,
                       ifelse(Output$logFC> 1 ,'Up','Down'),
                       'NoSignificant') #卡阈值
gene<-subset(Output,Output$change!='NoSignificant')#提取差异基因
write.csv(gene,"LIHC_diffgene.csv")
```
